# Effects of novel muscarinic M3 receptor ligand C1213 in pulmonary arterial hypertension models

**DOI:** 10.14814/phy2.13069

**Published:** 2016-12-30

**Authors:** Mohamed Ahmed, Sonya VanPatten, Satyan Lakshminrusimha, Hardik Patel, Thomas R. Coleman, Yousef Al‐Abed

**Affiliations:** ^1^ Northwell HealthPediatrics Department ‐ Neonatology DivisionCohens Children's Medical CenterManhassetNew York; ^2^ Department of Medicinal ChemistryCenter for Molecular InnovationManhassetNew York; ^3^ Departments of PediatricsPhysiology and BiophysicsState University of New York at BuffaloBuffaloNew York; ^4^ The Feinstein Institute for Medical ResearchManhassetNew York

**Keywords:** C1213, muscarinic receptor, nitric oxide, PPHN, pulmonary hypertension

## Abstract

Pulmonary hypertension (PH) is a complex disease comprising a pathologic remodeling and thickening of the pulmonary vessels causing an after load on the right heart ventricle that can result in ventricular failure. Triggered by oxidative stress, episodes of hypoxia, and other undetermined causes, PH is associated with poor outcomes and a high rate of morbidity. In the neonate, this disease has a similar etiology but is further complicated by the transition to breathing after birth, which requires a reduction in vascular resistance. Persistent pulmonary hypertension of the newborn (PPHN) is one form of PH that is frequently unresponsive to current therapies including inhaled nitric oxide (due to lack of proper absorption and diffusion), and other therapeutics targeting signaling mediators in vascular endothelium and smooth muscle. The need for novel agents, which target distinct pathways in pulmonary hypertension, remains. Herein, we investigated the therapeutic effects of novel muscarinic receptor ligand C1213 in models of PH. We demonstrated that via M3 muscarinic receptors, C1213 induced activating‐ eNOS phosphorylation (serine‐1177), which is known to lead to nitric oxide (NO) production in endothelial cells. Using signaling pathway inhibitors, we discovered that AKT and calcium signaling contributed to eNOS phosphorylation induced by C1213. As expected for an eNOS‐stimulating agent, in ex vivo and in vivo models, C1213 triggered pulmonary vasodilation and induced both pulmonary artery and systemic blood pressure reductions demonstrating its potential value in PH and PPHN. In brief, this proof‐of‐concept study provides evidence that an M3 muscarinic receptor functionally selective ligand stimulates downstream pathways leading to antihypertensive effects using in vitro, ex vivo, and in vivo models of PH.

## Introduction

Pulmonary hypertension (PH) is a chronic debilitating disease that elicits vascular remodeling, progressive hypoxemia, and right ventricular heart failure. Hypoxia leads to pulmonary vessel constriction, and persistent hypoxia causes uncontrolled proliferation of endothelial cells, smooth muscle cells (SMC), and adventitial fibroblasts. Each of these sequelae contribute to pulmonary vascular remodeling (vessel narrowing), which in turn chronically increases resistance to blood flow through the pulmonary circulation, leading to right ventricular failure and declining cardiac output.(Leopold and Maron 2016).

Vascular endothelial and smooth muscle cell interactions and downstream signaling pathways, including those induced by acetylcholine receptors (muscarinic), adrenergic receptors (Faber et al. 2007), and nitric oxide (NO) (Kysela and Torok 2000), are among the factors that are crucial to the regulation of vascular tone and therefore serve as therapeutic targets for interference with PH development and progression. Persistent pulmonary hypertension of the newborn (PPHN) is a disease characterized by resistance to therapies with a high rate of morbidity and mortality. The only currently FDA‐approved treatment for PPHN is inhaled NO (iNO) to which one third of patients may be resistant. Although other off‐label usage drugs are employed, including oral pulmonary vasodilators and phosphodiesterase inhibitors, there are not yet enough clinical studies to fully evaluate their safety and effectiveness (Lakshminrusimha et al., 2016a), leaving the search for agents which improve PH in the newborn an imperative and open field. Agents which stimulate endogenous NO production are also an intriguing avenue for treatment, as the release of NO from endothelial cells is known to activate soluble guanylate cyclase (sGC) in vascular smooth muscle cells which, through cyclic GMP production (cGMP) and Ca+2 efflux, leads to pulmonary vasodilatory responses (reviewed in (Tonelli et al. 2013; Quillon et al. 2015)). We began our project by searching our chemical library for agents that enhanced eNOS phosphorylation as this provided a surrogate measure of localized endogenous NO production. This screen resulted in the discovery of compound 1213.

**Figure 1:**
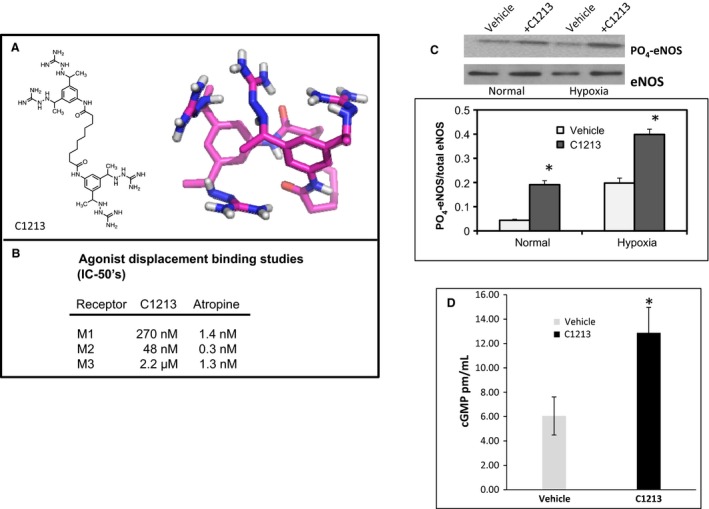
(A) Two‐ and three‐dimensional structure rendering of C1213 generated using Chemdraw Ultra (version 12.0.3.1216) and Molecular Operating Environment (version 2015.10). (B) Agonist‐binding displacement studies (functional antagonism) in Novascreen cells (Caliper‐Perkin Elmer) overexpressing muscarinic receptors. IC‐50 values are shown for C1213 versus no‐specific muscarinic antagonist atropine. (C) Phosphorylated eNOS protein expression normalized to total eNOS in HPMEC‐ST1.6R cells exposed to normal or hypoxic conditions and vehicle versus C1213 at 10 *μ*M. Experiment was repeated 3× on different days and representative blots are shown for indicated treatment/staining. * *P* < 0.05 compared with vehicle control. (D) cGMP expression in HPMEC‐ST1.6R cells exposed to vehicle versus C1213. * *P* < 0.05 compared with vehicle control.

Compound 1213 (C1213) is a tetravalent guanylhydrazone (Fig. 1A) which has been independently tested and shown to bind neurohumoral transmitter receptors including adrenergic receptors (*α*1 nonselective *K*_d_ = 2 × 10^−10^, *α*2 nonselective *K*_d_ = 1.5 × 10^−9^), dopamine receptors (D1 *K*_d_ = 1 × 10^−9^, D2 *K*_d_ = 7 × 10^−10^), opioid receptors (nonselective *K*_d_ = 2 × 10^−9^, ORL‐1 *K*_d_ = 5 × 10^−10^), a serotonin transporter (*K*_d_ = 1.7 × 10^−9^), a histamine receptor (H2 *K*_d_ = 7 × 10^−10^), and muscarinic receptors (nonselective *K*_d_ = 1 × 10^−10^). More specific testing for functional agonism and antagonism was then carried out for the muscarinic acetylcholine receptors as they are known to play crucial roles in pulmonary artery vasoconstriction/vasodilation (Orii et al. 2010; Harvey 2012), with species‐specific differences noted (Walch et al. 1999). M3 muscarinic receptors are prevalent in both pulmonary vascular smooth muscle (Walch et al. 2001), and have been shown to mediate pulmonary artery vasodilatory responses to acetylcholine in an ex vivo model (Orii et al. 2010). In this report we set out to determine the effect of C1213 in in vitro, ex vivo, and in vivo PH and PPHN models. An agent that stimulates endogenous NO production, with functionally selective activity on muscarinic receptors could provide a unique means to interfere with PH‐driven pathologies.

## Material and Methods

All animal studies were performed in accordance with accepted guidelines and IACUC requirements. Data significance was measured using successive t‐tests with a *P*‐value of 0.05 as a cutoff. All chemicals and reagents were acquired from Sigma‐Aldrich (St. Louis, MO) or indicated vendors. Compound 1213 was synthesized by Dr. Al‐Abed and was ≥95% pure by HPLC and structure was confirmed by MS and NMR (structure illustrated in Fig. 1). Cell‐line HPMEC‐ST1.6R was negative for mycoplasma contamination upon receipt.

### In vitro endothelial cell studies

#### Neurotransmitter/neurohumoral receptor assays

Binding (K_d_'s), and functional agonist, and antagonist (calcium mobilization) assays were performed by NovaScreen Biosciences (currently part of Caliper‐Perkin Elmer, Hopkinton, MA) as a fee for service by standardized protocols using cell lines overexpressing respective receptors under investigation.

#### Endothelial cell culture and hypoxia exposure

The human pulmonary microvascular endothelial cell line, HPMEC‐ST1.6R, was kindly provided by C. James Kirkpatrick, Institute of Pathology, Johannes‐Gutenberg University, Germany. This cell line was generated by cotransfection with pSV3neo and pC1.neo.hTERT (Krump‐Konvalinkova et al. 2001). At least 99% of cells were endothelial cells as evidenced by staining for platelet endothelial cell adhesion molecule (PECAM‐1, CD31), von Willebrand factor (vWF), and the adhesion molecules, intercellular adhesion molecule (ICAM‐1), vascular adhesion molecule (VCAM‐1), and E‐selectin. In addition, HPMEC‐ST1.6R showed an angiogenic response on Matrigel similar to that of primary HPMEC and was not tumorgenic in nude mice (Krump‐Konvalinkova et al. 2001). Endothelial cells (ECs) were cultured using Endothelial Cell Growth Medium (C22020 Heidelberg, Germany) under 5% CO_2_ at 37°C. Media was changed every second day. Hypoxia was created by flushing the chamber with 95% Nitrogen and 5% CO_2_ gas mixture. Cells were incubated under hypoxia for 1 h.

#### endothelial Nitric Oxide Synthetase (eNOS) phosphorylation assay

Compound 1213 was added to EC culture immediately before hypoxia exposure. One hour later, phosphorylation of serine‐1177 endothelial nitric oxide synthetase (eNOS) was measured by Western blot in both ECs incubated in room air (RA) or exposed to hypoxia. Commercially available antibodies for serine‐1177 PO_4_ eNOS, total eNOS (Cell Signaling Technologies Danver, MA), and secondary antibodies (Santa Cruz Biotechnologies, Santa Cruz, CA) were used. Cells were washed with ice‐cold PBS twice and were immediately lysed in the plate using RIPA buffer supplemented with protease and phosphatase inhibitor cocktail (Roche Applied Science, Indianapolis, IN). Proteins were resolved on SDS‐PAGE and blotted on PVDF membrane. Beta‐actin (Ab from Genscript, Piscataway, NJ) was used as a loading control for normalization. Different doses of C1213 were used to assess target dose in relation to maximum physiological function and 10 *μ*M was selected for studies. Densitometry was performed using Image J software which is freely available at https://imagej.nih.gov/ij/index.html. cGMP as a product of NO production was also assayed in EC culture cells after treating them with vehicle or C1213 (ELIZA assay, Cayman, Ann Arbor, Michigan).

#### Acetylcholine receptor blockade study

We used a series of acetylcholine receptor blockers and measured (serine‐1177) phosphorylated eNOS protein (normalized to actin) as a read out. Both C1213 (10 *μ*mol/L) and indicated blockers (antagonists) were added to EC tissue culture and the western experiment described above was repeated. The following receptor antagonists were used: Atropine at dose of 0.01 *μ*mol/L was used as nonspecific muscarinic (M) receptor blockade, pirenzepine (M1 blocker) at doses 1,10 and 100 *μ*mol/L; dareifenacin (M3 blocker) at doses 0.01, 0.1, and 1 *μ*mol/L, and methyllycaconitine (MLA) nicotinic blocker at doses 0.1, 1, and 10 *μ*mol/L. Pirenzepine dihydrochloride was purchased from Tocris Bioscience (Bristol, UK).

#### Signaling pathway studies

To investigate downstream signaling events, the following kinase inhibitors were employed: wortmannin (upstream inhibitor of Akt), dorsomorphin dihydrochloride aka “compound C” (AMPK inhibitor), and BAPTA‐AM (Ca+2 chelator). Wortmannin, compound C, and BAPTA‐AM were purchased from Tocris Bioscience (Bristol, UK). Western blot for AKT, pAKT, and actin was performed as described above. Commercially available primary antibodies for AKT, pAKT, and secondary antibodies were used in westerns (Santa Cruz Biotechnologies, Santa Cruz, CA).

### Ex vivo pulmonary artery studies

Physiological function assessments were performed on explanted main intralobar pulmonary arteries with intact endotheliumas previously described in mice (Ahmed et al. 2012). The pulmonary arterial rings (experiments were repeated five times using five rings) were contracted with increasing cumulative doses of phenylephrine (PE) (1, 10, and 100 nmol/L) doses followed by relaxation with increasing doses of acetylcholine (Ach) (10 nmol/L, 100 nmol/L, 1 mmol/L, and 10 mmol/L) (Ach–8 to Ach–5). Arteries were again reequilibrated with normal Krebs‐bicarbonate buffer for another 30 min and then were contracted with 100 nmol/L phenylephrine followed by a dose of C1213 compound (10 *μ*M).

### In vivo studies—PPNH model

This study was approved by the Laboratory Animal Care Committee at the State University of New York (SUNY) Buffalo. Fetal ductal ligation was performed 20 days preterm at 125 days of gestation (*n* = 2) in time‐dated pregnant sheep (Morin 1989). After a 9‐d recovery, lambs with PPHN were delivered by cesarean section. Alive lambs were intubated, and standard of care equivalent for human neonates was followed (Lakshminrusimha et al. 2006). Thoracotomy and instrumentation of the pulmonary arteries (PA) was achieved and oxygenation was measured as arterial‐to‐alveolar oxygen ratios: a/A ratio = umbilical arterial PaO_2_/[745 ‐ 47] X FiO_2_ ‐ PaCO_2_/0.8, where 745 mmHg is the barometric pressure at Buffalo, and 47 mmHg is the partial pressure of water. Pulmonary vascular resistance (PVR) was calculated using the following equation: PVR (mmHg.mL^−1^.min.kg body weight) = (mean PA pressure‐left atrial pressure in mm Hg) 47 pulmonary blood flow corrected for body weight (*Q*_p_, mL.min^−1 ^kg^−1^). Systemic and pulmonary pressure, pulmonary and coronary blood flow, PaO_2_, and PaCO_2_ were recorded and once the animal was stable (about 2 h post partum), an IV bolus dose of 1213 (4 mg/kg – approx. 80 *μ*mol/L in blood max.) was given. Readings of systemic and pulmonary vascular pressure and PO_2_/PCO_2_ were monitored postinjection of C1213.

### Statistical analysis

Statistical evaluation of eNOS expression using different blockers was performed using the Analysis of Variance test (ANOVA). T‐test was used in comparison between two groups. A *P*‐value of less than 0.05 is considered to be significant. Statistical software SPSS Version 17 and MS Excel were used to analyze the data.

## Results

### C1213 Functional receptor activation studies

Neurohumoral/neurotransmitter receptor functional activation was explored using a Novascreen G‐protein coupled receptor (GPCR) calcium flux assay (Caliper‐ Perkin Elmer, Hopkinton, MA). For agonist studies, C1213 (2D and 3D structure shown in Fig. 1A) was added at a range of doses and Ca+2 mobilization was measured. No agonistic activity was observed for the receptors studied (data not shown) at lower doses of compound (nanomolar range). At the highest two concentrations tested (5 and 10 *μ*mol/L) in cells overexpressing certain receptors, C1213 induced nonspecific calcium flux through an unelucidated mechanism of action. This finding is difficult to interpret as these cells are engineered (proprietary) to stimulate calcium release as a readout of GPCR activation and also express much higher levels of receptors than a native cell, alternatively, compound solubility issues in the specialized buffer may have been the cause.

For functional antagonism studies, we found that C1213 was able to inhibit agonist (acetylcholine) stimulation of calcium flux for muscarinic receptors M1, M2, and M3, with IC‐50's shown in Figure 1B compared with nonspecific muscarinic antagonist atropine.

#### Effect of C1213 in hypoxia versus room air

Our data demonstrated that hypoxia led to an increase in the serine‐1177 eNOS phosphorylation as expected. Addition of C1213 for 1 h elicited a significant induction of ser‐1177 eNOS phosphorylation in the endothelial cell‐line HPMEC‐ST1.6R in both normal (room air, FiO2 21%) and hypoxic (FiO2 1% for 60 min) conditions (Fig. 1C). To support our findings, cGMP was also measured and showed marked significant increase in NO production and its bioavailability, marked by significant increase in cGMP in treated EC culture with C1213 (Fig. 1D). These data strongly suggest that C1213 will meaningfully augment endogenous NO production (Barauna et al. 2013; Tonelli et al. 2013).

#### Acetylcholine receptor antagonism studies in pulmonary endothelial cells

**Figure 2:**
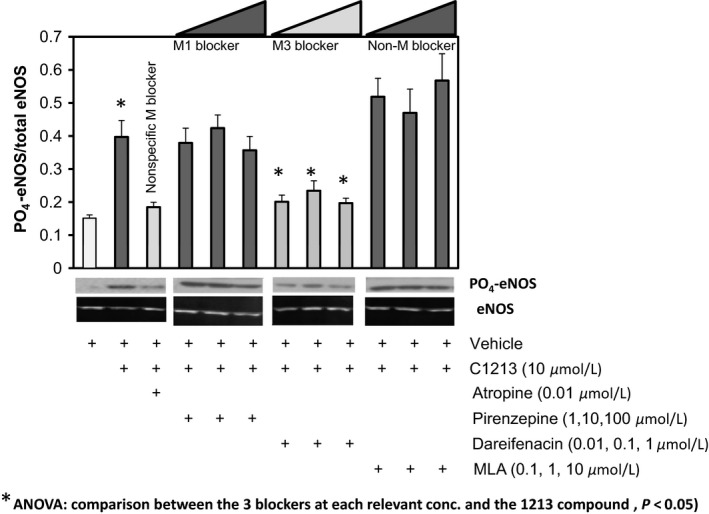
Total eNOS normalized phosphorylated eNOS expression in HPMEC‐ST1.6R cells exposed to acetylcholine receptor blockers at doses sequentially indicated. Experiment was repeated 5× on different days and representative blots are shown for indicated treatments/staining.

These studies investigate the specific neurohumoral receptor subtype responsible for C1213 effects on serine‐1177 eNOS phosphorylation, which is a known stimulator of eNOS activity (Kolluru et al. 2010). In standard conditions, C1213 significantly increased the relative abundance of phosphorylated eNOS protein compared with total eNOS (Fig. 2). In contrast, the coaddition of atropine (a nonspecific competitive antagonist of muscarinic receptors) essentially blocked the 1213‐mediated increase in phosphorylated eNOS. As atropine acts on all muscarinic receptors (M1 through M5), we next used more specific receptor antagonists. Coaddition of pirenzepine (an M1 blocker) did not impact C1213 activity, whereas dareifenacin (a specific M3 blocker) markedly reduced C1213‐mediated eNOS phosphorylation (Fig. 2). In a control experiment, coaddition of methyllycaconitine (MLA, a nicotinic acetylcholine receptor blocker that does not exert its effects through muscarinic receptors) caused no meaningful change in eNOS phosphorylation (Fig. 2). Taken together, these findings suggest that C1213 will stimulate endogenous NO production through eNOS activation in pulmonary vascular endothelial cells and this stimulation is mediated specifically through M3 muscarinic receptors.

#### Downstream signaling studies

**Figure 3:**
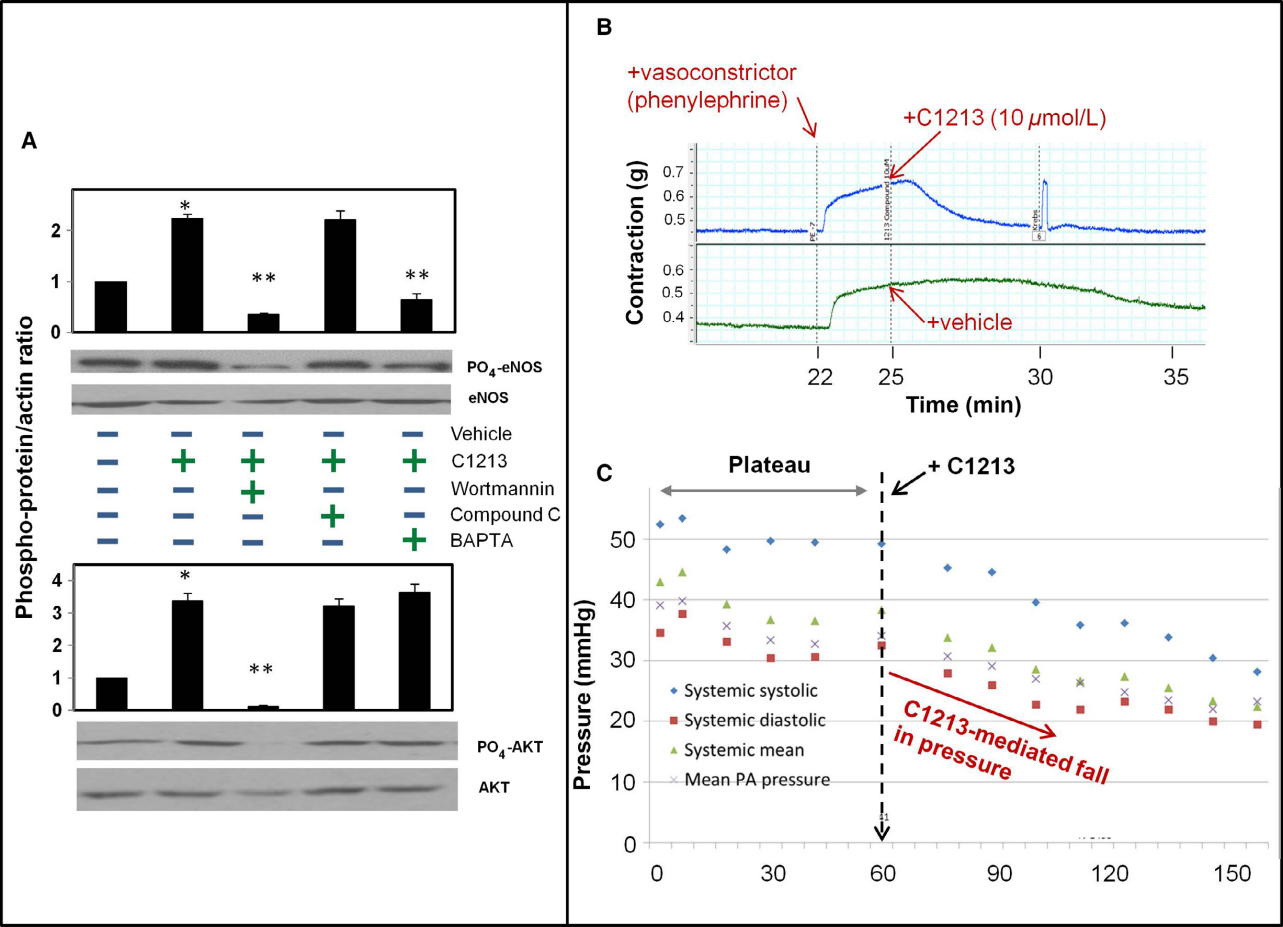
(A) Phospho‐protein expression normalized in HPMEC‐ST1.6R cells exposed to kinase inhibitors and calcium blocker (top panel—normalized to eNOS; bottom panel—normalized to AKT). Experiment was repeated 3× on different days and representative blots are shown for indicated treatments/staining. ANOVA was used to compare effect of each individual blocker, vehicle, and 1213 compound in expression of p‐eNOS or p‐AKT; * *P* < 0.05. (B) Vascular contraction studies in excised murine endothelium‐associated pulmonary arteries. After arteries were tested for responses, baseline was stabilized and phenylephrine was infused (22 min), followed by C1213 (top panel—blue trace) or vehicle (bottom panel—green trace) at 25 min (*n* = 5 arterial rings). (C) Systemic and pulmonary artery blood pressure responses to C1213 infusion (dashed vertical line) of 4 mg/kg in neonatal hypertension model (*N* = 2 animals).

Protein kinases (AKT and AMPK) activate NO production through phosphorylation of eNOS at specific amino acid residues. We investigated the functional involvement of AKT‐ and AMPK‐dependent and ‐independent pathways in C1213‐induced eNOS phosphorylation (ser‐1177) by looking at cell lysates of pulmonary endothelial cell cultures treated with C1213 for 1 h, in the presence and absence of various kinase inhibitors. When coincubated with wortmannin (AKT inhibitor) or BAPTA (Ca+2 chelator), eNOS phosphorylation was dampened, however, compound C (AMPK inhibitor) had no effect (Fig. 3A). The relative downregulation of eNOS phosphorylation in the presence of these kinase inhibitors suggests that C1213 mediates eNOS phosphorylation by activating AKT and/or through Ca+2‐dependent means (likely via CaMKII) (Fig. 3A) (*P* < 0.05; ANOVA; comparing effect of each blocker to vehicle and 1213 compound on p‐eNOS/eNOS ratio). We next examined the effect of C1213 on AKT activation by assessing its phosphorylation state in the presence of the same kinase inhibitors. When normalized to total AKT, C1213 induced AKT phosphorylation and as anticipated, only wortmannin profoundly inhibited AKT phosphorylation (Fig. 3A, lower panel) (ANOVA test; *P* < 0.05). Taken together, these finding begin to decipher pathways by which C1213 stimulates NO production: it increases the phosphorylation and activation of eNOS mainly through AKT and Ca+2 signaling mechanisms, but not through AMPK.

#### Ex vivo studies

In our mice studies of explanted PA vessels with endothelium, we found that C1213 (10 *μ*mol/L) rapidly (within 2 min) overcame the contraction induced by PE and the tone essentially dropped to the pre‐PE baseline level within 1–2 min (Fig. 3B). Thus, C1213 rapidly relaxes contracted pulmonary vasculature when compared with vehicle control.

### In vivo studies

For our studies we used the gold standard model of PPHN in lambs, where PH is induced by ductal ligation in utero. The success of our model of PPHN was demonstrated by a peak PA pressure of close to 40 mmHg (a normal range for PA pressure is 15–20 mmHg). In the initial phase of monitoring, systemic and PA pressures and ventilation status were stabilized allowing for acclimation (Fig. 3C—plateau). Systemic (IV) administration of C1213 induced a marked (~25%) drop in both PA and systemic (systolic and diastolic) pressures (Fig. 3C—post‐C1213 dashed vertical line). Effects of C1213 on oxygenation and ventilation are also of note. Administration of C1213 induced an increase in PaCO_2_ and complementary decrease in PaO_2_. At 114 min, this pattern was reversed: PaCO2 precipitously fell and PaO2 precipitously rose. These changes were likely governed by the C1213‐mediated decrease in PA flow. Focusing on the critical variable of oxygenation, C1213 causes the mean PA pressure to fall, when it goes below ~30 mmHg (which takes ~40 min) a rapid reflexive increase in PaO2 and corresponding decrease in PaCO2 is elicited which then normalizes.

## Discussion

**Figure 4:**
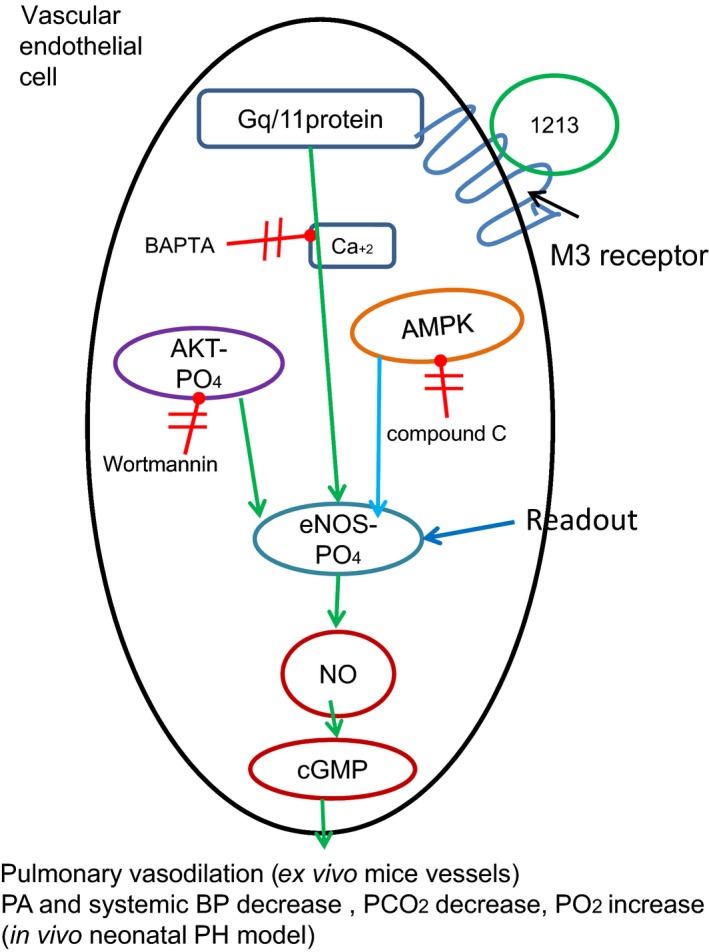
Summary of the main effects of M3 receptor ligand C1213 on endothelial signaling pathways involved in pulmonary vasodilation and pulmonary arterial hypertension.

Although O_2_ and NO inhalation therapy remains some of the most effective treatments for PPHN, this research has revealed the risks of oxidative stress that can be induced by inhaled oxygen (and potentially iNO) therapies (reviewed in [Lakshminrusimha et al., 2016b]). Evidence that oxidative stress is a crucial factor in PPHN (Afolayan et al. 2012, 2016), as well as cases refractory to iNO therapy, provided the basis for our search for more moderate ways to stimulate the endogenous NO response (EC eNOS>NO>SMC sGC>cGMP>Ca+2 > SMC relaxation) as a means to modulate PPHN. A screen for eNOS activators (serine‐1177 PO_4_) lead to the discovery of C1213, which was found to act as a functionally selective agonist on M3 receptors of pulmonary vascular endothelial cells. A summary of our main findings in endothelial cells and ex vivo and in vivo models of PPHN is presented in Figure 4.

Combined, our results provide evidence for the continued investigation of C1213 as a new experimental vasodilatory agent, capable of stimulating endogenous NO production from endothelial vascular cells, leading to relaxation of smooth muscle pulmonary vasculature, and reduction in pulmonary hypertension. Additional testing indicated that C1213 may act as either a competitive antagonist or functionally selective agonist on muscarinic receptors, which was dependent on the downstream assay monitored and/or cell type used for study. Future studies will look at larger sample size of animal model with PPHN and look at the longer‐term effects of C1213 administration and help clarify other potential mechanisms (modulation of other neurohormonal receptors or mediators) by which C1213 generates its effects on pulmonary arterial and systemic vascular pressure.

## Conflict of Interest

None declared.
